# Influence of laser beam profile on morphology and optical properties of silicon nanoparticles formed by laser ablation in liquid

**DOI:** 10.3762/bjnano.16.108

**Published:** 2025-09-04

**Authors:** Natalie Tarasenka, Vladislav Kornev, Alena Nevar, Nikolai Tarasenko

**Affiliations:** 1 B. I. Stepanov Institute of Physics, National Academy of Sciences of Belarus, 220072 Minsk, Belarus; 2 Department of Design, Manufacturing and Engineering Management, University of Strathclyde, Glasgow, UK

**Keywords:** laser ablation, laser beams with Bessel, annular, Gaussian profiles, laser-induced modification, silicon nanoparticles

## Abstract

In this study, silicon nanoparticles (NPs) were produced by pulsed laser ablation in a liquid, aiming to investigate the influence of a laser beam profile on the properties of the resultant NPs. Morphology, inner structure, and phase composition of the formed NPs were characterized by means of ultraviolet–visible spectroscopy, high-resolution transmission electron microscopy, and Raman and photoluminescence spectroscopies, and the correlation of the NP properties with the laser beam profile was studied. Three different beam profiles were selected, namely, a Bessel beam produced using an axicon, an annular profile formed using a combination of an axicon and a converging lens, and a Gaussian beam focused on the surface of a Si target using the same converging lens. In all the schemes, a nanosecond Nd^3+^:YAG laser with a pulse duration of 10 ns operating at its fundamental harmonic (1064 nm) was used as an ablation source. The beam profile has been shown to be a crucial factor significantly influencing morphology and composition of the nanostructures produced. Namely, the conditions generated using a Bessel beam profile favored the production of nanostructures having elongated filament-like morphology. The synthesized colloidal Si NPs are suggested for applications as a component of electrode materials in supercapacitors and batteries.

## Introduction

Silicon, due to its prominent optical and electronic properties, is the most widely used semiconductor material in many fields of microelectronics, optics, photovoltaics, and molecular sensing. Nevertheless, even despite its widespread application, the interest in silicon-based structures, especially having sizes in the nanometer range and different morphologies is continuously growing. One aspect of current studies is further improvement of the properties and performance of the developed silicon-based devices. As a result, crystalline films based on silicon [1–2] as well as nano-sized silicon materials have become an emerging field of research [3–7]. The expanding application areas require silicon nanomaterials of different morphologies, including quantum dots, nanowires, and nanocrystalline films. Si nanowires, for example, are considered as a promising alternative to silicon electrodes in Li-ion batteries by providing a solution to the urgent problem of large volumetric expansion known for Si during lithiation process [3]. Due to the possibility of lateral expansion in Si nanowires, the cracking of the electrodes during lithiation is minimized. In addition, the one-dimensional structure of Si nanowires facilitates the electronic transport along the wire axis, while the transport of the ions occurs in the transverse direction, providing shorter diffusion distances. All these aspects underline the benefits of Si nanowires and the need to develop novel approaches for their formation.

Despite certain progress in this field, there is still a need for reliable and controlled synthesis methods for silicon-based nanostructures. Nowadays, pulsed laser ablation in liquids (PLAL) has been recognized as a general and important route for the synthesis of nanoparticles (NPs) with tuned optical and electrical properties suitable for a wide range of practical applications [7–9]. PLAL has a number of advantages over other NP production methods due to the accessibility of equipment for ablation, the simplicity of the method, which does not require a special environment such as vacuum, and the ability to alter the properties and morphology of NPs by changing the laser parameters and liquid media [10–11]. The control over the major characteristics of the produced nanomaterials can be achieved by adjustment of several experimental parameters, for example, laser wavelength, fluence, pulse width, repetition rate, and liquid composition. The parameters of the ablated target, such as its optical characteristics and conductivity/resistivity, might also have a pronounced impact on size and morphology of the produced NPs. As demonstrated in [12], femtosecond laser ablation of silicon with varying conductivities resulted in NPs of different morphology, structure, and optical characteristics.

It should be noted that, as a rule, NPs of nearly spherical morphology are formed by PLAL under typical experimental conditions. Non-spherical NPs were also reported to be produced by PLAL [13]; but this requires special configurations of laser ablation experiments, such as laser ablation in an applied external electrical field [14–16], variation of liquid composition [13], laser ablation at increased liquid temperature [17–18], and using different light polarization (linear or circular) [19]. As a result, a number of approaches have been adopted to the formation of semiconducting nanowires and hybrid nanostructures by PLAL, including one-dimensional nanowires, nanotubes, and nanoribbons, as well as two-dimensional nanosheets and nanobelts [13,20–21]. Exemplary applications of laser ablation techniques for the preparation of non-spherical NPs are the fabrication of semiconductor nanowires such as hybrid CdS [20] and ZnS nanowires [21]. Si nanowires can also be prepared by laser ablation [4–5]. However, the synthesis is typically performed in gaseous atmosphere, and the developed approaches require high temperature [5,22] or pressure [23], or the presence of a catalyst [24]. Consequently, alternative approaches for the formation of Si nanowires by laser ablation in liquid environment and under ambient conditions are of high interest. In this work, we have applied a method based on the change of the spatial laser beam energy distribution (Gauss, Bessel, or annular) for the synthesis of silicon nanomaterials of different shapes by laser ablation in a liquid. The advantages of laser beams with Bessel and annular profiles were demonstrated earlier in the fields of material processing, such as microdrilling, fabrication of micro/nano channels in transparent materials, nanopatterning, and photopolymerization [25–26]. The achieved advances in these fields allow one to expect benefits of varying beam shapes for laser ablation synthesis in liquids as well. However, studies that use different beam shapes in laser ablation synthesis of nanomaterials in liquids are still very rare. A typical PLAL experiment uses a Gaussian beam profile, which is known to have a tight focus along both transverse and propagation axes and the highest power density in the beam center. Non-Gaussian beam profiles are characterized by a non-uniform energy distribution, which will affect plasma generation and confinement, the hydrodynamic trajectory of the ejected target material and pressure relaxation, as well as plasma and cavitation bubble propagation and temporal evolution. In the case of a Bessel beam, the focusing with an axicon provides longer depth of focus with a sharp central intensity, which is surrounded by concentric rings. In the case of annular beam ablation, the characteristic feature is the formation of a stagnation layer at the center of the plasma normal to the target, which results in a plasma jet near the target surface [27] with different plasma parameters than in the plasma generated by a Gaussian beam.

A change of the incident beam pattern will change the temperature and pressure inside cavitation bubbles (CBs) and influence CB oscillations. Furthermore, pressure variations at the target interface would be induced, which is a substantial factor determining the morphology of the forming nanomaterials. As a result, the conditions created by different beam shapes might favor the formation of non-spherical NPs, nanoporous alloys, and hierarchical nanostructures. This is important to extend the application areas of the synthesized products towards energy- and catalysis-related applications due to materials’ high surface area and prompt reaction kinetics. The potential benefits of the beam shape variation have recently initiated more active research on the ablative generation of NPs using spatially shaped laser pulses. For example, laser beams with a donut shape were used for ablating gold, yttrium oxide, and high-entropy alloy targets in water. The donut beam enabled NP size reduction and narrower size distributions [28]. Application of femtosecond Bessel beams for the ablation of silver in a liquid was reported in [29], motivated by the possibility to use the advantages of higher depth of focus and invariance of the core intensity profile with propagation for NP production. However, the NPs synthesized in [28–29] had a quasi-spherical shape; therefore, an influence of the beam profile on the morphology of the NPs in comparison with usually applied Gaussian laser beams was not discussed. To the best of our knowledge, not much attention has been paid to the influence of different spatial laser beam profiles on the shape of the nanostructures formed in the process of laser ablation. Among the rare reports on non-spherical nanostructures synthesized using different beam shapes is a work by Marappu et al [30], where a cylindrical focusing geometry was demonstrated to result in the fabrication of silver nanoribbons via picosecond laser ablation of bulk silver in doubly distilled water.

Therefore, studies aiming to provide insights on how the laser beam profile influences the morphology of NPs produced by PLAL are of high importance to go beyond the typically observed spherical NPs. Our work aims to investigate the influence of the laser beam profile on the properties of silicon NPs produced by PLAL. The formation of Si nanostructures with elongated filament-like shape is demonstrated for the first time using laser ablation in ethanol with a Bessel beam. Furthermore, we demonstrate a preparation of Si NPs with different morphology using Bessel, annular, and Gaussian laser beams. We believe that the obtained results are important for the preparation of silicon-based nanocomposites as electrode materials with enhanced performance.

## Materials and Methods

**Figure 1:**
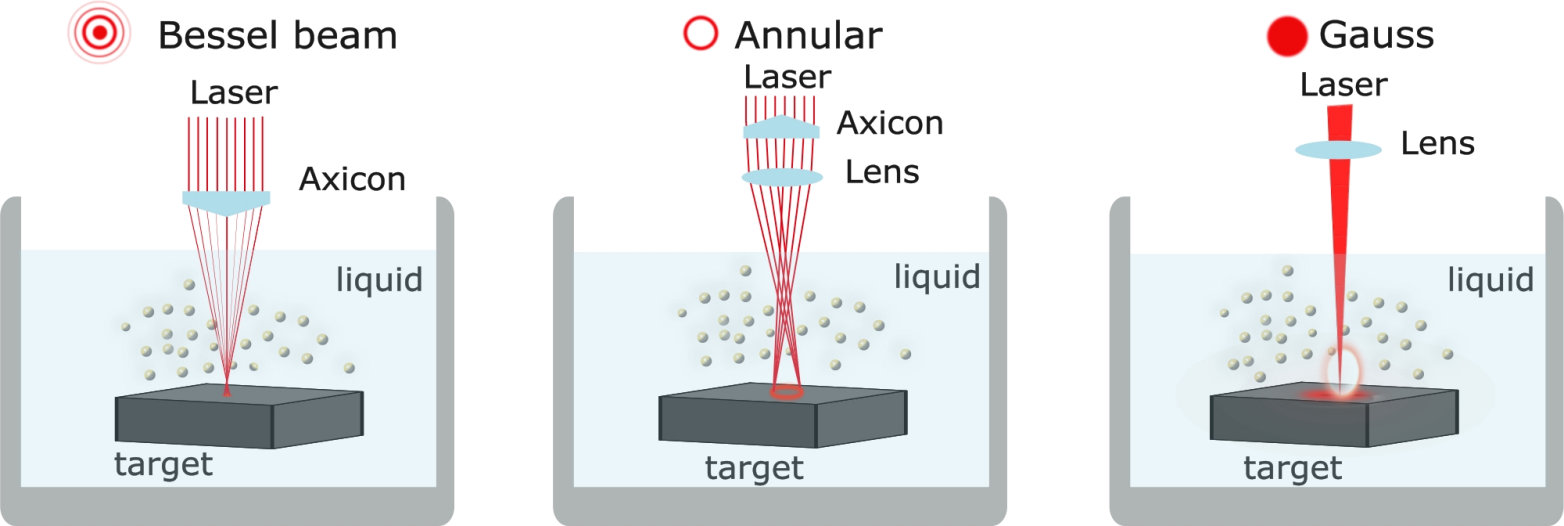
Schemes of laser ablation using Bessel (a), annular (b) and Gaussian (c) profiles.

The laser ablation experiments were performed using a nanosecond Nd:YAG laser (LS2131D, LOTIS TII, Belarus) as an excitation source, operating at 1064 nm (energy 80 mJ per pulse, repetition rate 10 Hz, pulse duration 10 ns, laser beam diameter 6 mm, ablation time 20 min) in a double-pulse mode with an interpulse delay of 1 μs [7,10]. These parameters were maintained for all three cases of focusing optics forming spatial profiles of Bessel, annular, and Gaussian beams. As a target, a silicon plate (boron-doped Si wafer with resistivity 12 Ω·cm) was used, which was fixed in a holder and placed at the bottom of a 15 mL glass beaker (Figure 1) under an ethanol layer in a way that a 3 mm distance was kept between the surface of the liquid and the target. A Si target was constantly moved with a micrometer movement system during laser ablation. For this, the beaker was placed on the *xy*-motorized stage to ablate the target while scanning with a speed of 2.7 mm·s^−1^ from left to right and back. The scanning region was 12 × 6 mm with a hatch spacing of 0.6 mm. Ethanol as a working liquid minimized oxidation and carburization of the formed NPs.

Laser ablation was performed using three different spatial profiles Bessel (Figure 1a), annular (Figure 1b), and Gaussian (Figure 1c). The Bessel beam (Figure 1a) was produced using an axicon (axicon angle 7°), the annular profile was formed using a combination of the same axicon and a converging lens (*f* = 3 cm), and the Gaussian profile was formed using the same converging lens focusing the laser radiation on the surface of the Si target.

To illustrate the size and energy distribution of Gaussian and Bessel laser beams used in the experiments we have compared images of the laser spots on the silicon target ablated by different beams (Supporting Information File 1, Figure S5). The diameter of the ablated spot in case of a focused Gaussian beam was measured to be 415 μm as determined from the SEM analysis (Supporting Information File 1, Figure S5d). The resulting fluence on the target was estimated to be 118.4 J·cm^−2^.

A Bessel beam pattern was generated using a glass axicon with a base angle α = 7°, which was irradiated with a collimated Gaussian beam of a Nd:YAG laser. A Bessel zone formed ca. 70 mm away from the apex of the axicon, which is where the sample surface was placed. The resulting beam has a conical half-angle θ = 1.48° and a central core diameter of *r*_0_ = 20 μm (Supporting Information File 1, Figure S5). The θ angle is related to the base angle of the axicon and can be estimated from the formula θ = arcsin(*n*_0_·sin α) − α [31], where *n*_0_ is the refractive index of the axicon. The formed Bessel interference pattern is characterized by a superposition of a central core and concentric rings, which carry a fraction of the total pulse energy equal to 2*r*_0_/ω_0_ [32]. Accordingly, the peak fluence in the central lobe can be calculated as [31–32]:


[1] $$F_{\text{B}}=\frac{2r_{0}}{\omega_{0}}\frac{E_{\text{in}}}{\pi r_{0}^{2}}=\frac{2E_{\text{in}}}{\pi \omega_{0}r_{0}},$$

where *E*_in_ is the incident Gaussian pulse energy (80 mJ per pulse) and ω_0_ is e^−2^ radius of the incident Gaussian beam (3 mm). Following these calculations, the fluence was calculated to be 84.9 J·cm^−2^.

Morphology, phase composition, and structure of the formed NPs were analyzed by means of ultraviolet–visible (UV–vis) spectroscopy, high-resolution transmission electron microscopy (HRTEM), selected area electron diffraction (SAED), X-ray diffraction (XRD), and Raman and photoluminescence (PL) spectroscopies. Particle size and morphology were investigated by transmission electron microscopy (TEM) using a JEOL JEM-2100F (JEOL, USA) operating with an accelerating voltage of 200 kV. For TEM and SAED measurements, the NPs were drop-casted onto copper grids covered by carbon film and dried under ambient conditions. Crystalline structure, phase composition, and lattice parameters of the formed Si nanostructures were determined from HRTEM images by fast Fourier transformation (FFT). The morphology of the produced nanoparticles was also evaluated using scanning electron microscopy (SEM) with a SUPRA 55WDS microscope (Carl Zeiss, Germany) operating at an accelerating voltage of 10 kV. For SEM and Raman measurements, the colloidal solution was deposited onto Al foil by drop casting and dried at room temperature. The Raman measurements were performed under 488 nm excitation using a micro-Raman system (“NanoFlex”, Solar LS, Belarus). The UV–vis absorption spectra of the prepared colloids were measured using a Cary 500 Scan spectrophotometer (Varian, USA) in the spectral range of 200–2000 nm in a 10 mm quartz cuvette. The measurements of the PL spectra were carried out at room temperature using a Fluorolog-3 spectrofluorometer (HORIBA Scientific, USA) equipped with Peltier-cooled CCD-detector and with a xenon arc lamp for excitation. The measured spectra were corrected for the spectral sensitivity of the spectrofluorometer.

## Results and Discussion

### Morphology and crystal structure

**Figure 2:**
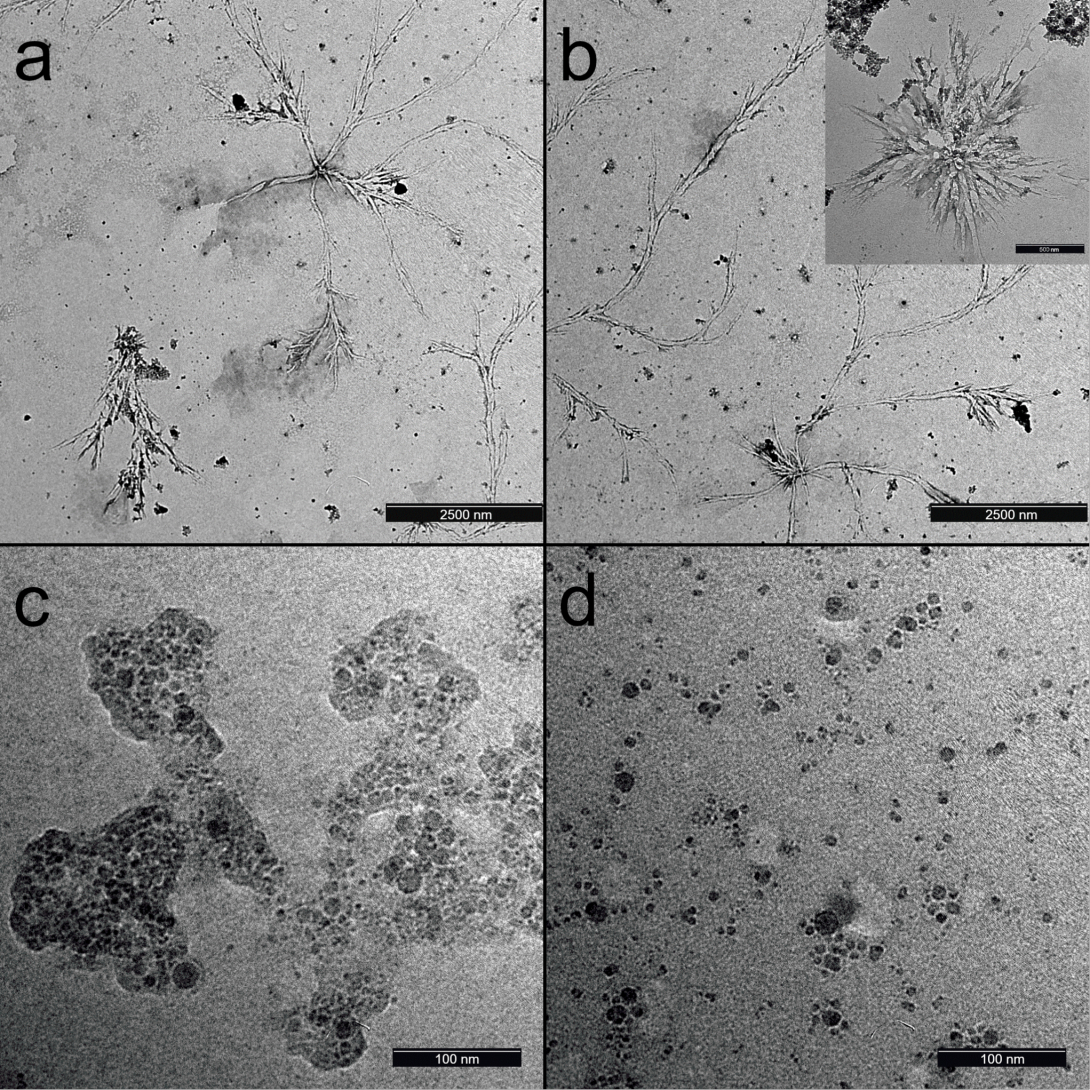
TEM images of Si nanoparticles prepared using (a, b) Bessel, (c) annular, and (d) Gaussian beams.

The morphology characterization of as-formed Si NPs was performed by TEM and SEM. Figure 2 shows TEM images of the Si NPs obtained by laser ablation in ethanol using Bessel (Figure 2a,b), annular (Figure 2c), and Gaussian (Figure 2d) laser beams. While Si NPs formed using annular and Gaussian profiles result in nearly spherical NPs, laser ablation with a Bessel beam resulted in the two types of nanostructures, namely, interconnected branched filament-like structures with a diameter of about 70 nm (Supporting Information File 1, Figure S3) and a length in the range up to several micrometers and small spherical nanocrystallites with diameters below 10 nm (Figure 2a,b). The sizes of the spherical Si NPs produced using Gaussian and annular laser beam profiles were 7.5 and 9.6 nm, respectively (Supporting Information File 1, Figure S2). However, NPs produced using the annular beam profile showed higher polydispersity than those produced with a Gaussian profile. The average size of the Si NPs was calculated using ImageJ software.

A more detailed analysis of the TEM images showed the tendency of the particles prepared using an annular beam to gather into aggregates surrounded by a non-uniform matrix or shell, while in the case of a Gaussian beam, the resulting NPs appear more separated with no outer shell observed. Mostly, the formation of spherical NPs with clear surface boundaries was observed, which is typical for laser ablation of semiconductors.

**Figure 3:**
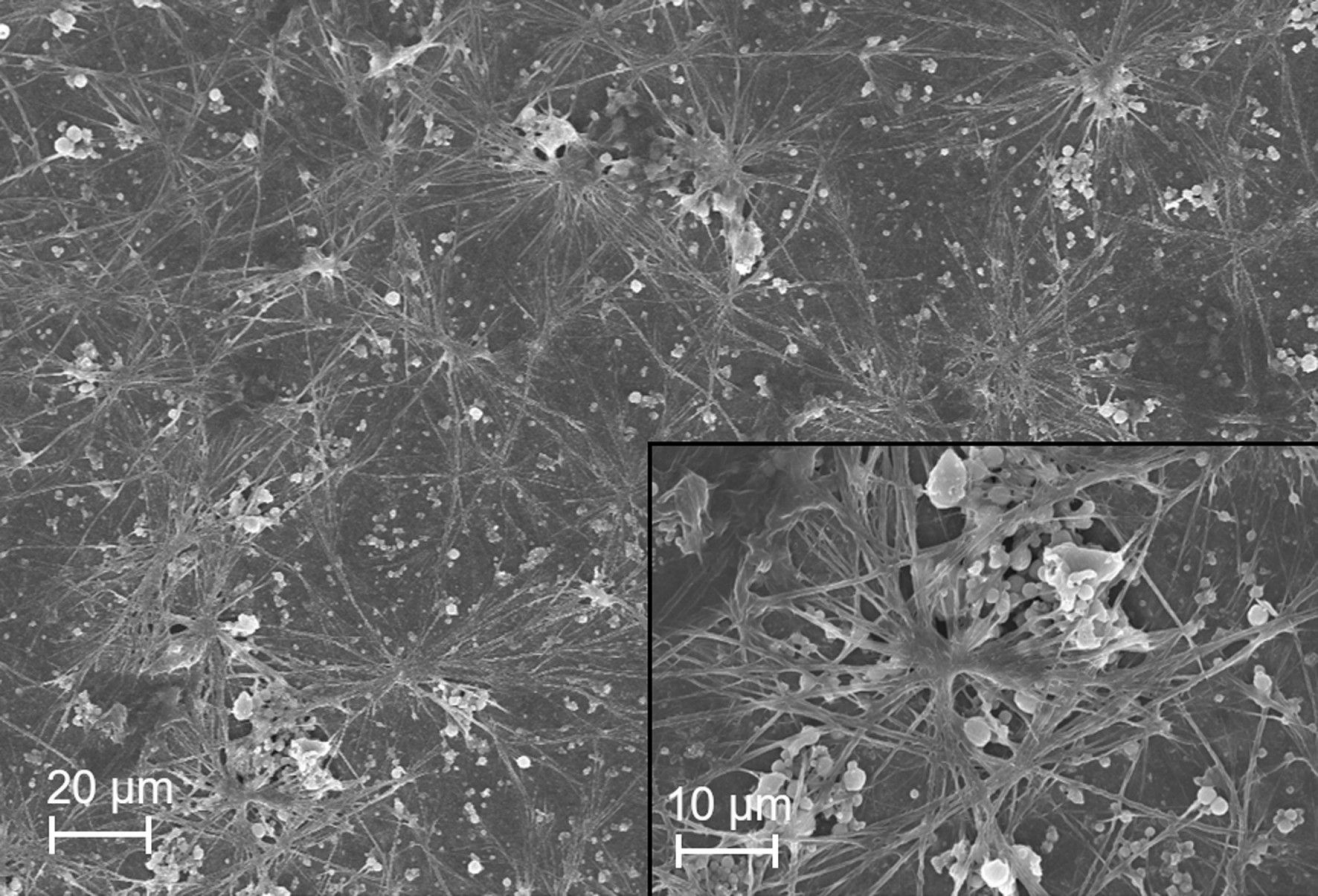
SEM images of the Si nanoparticles, prepared using the Bessel beam.

The interesting morphological peculiarity of the Si nanostructures produced with a Bessel beam was further confirmed by their SEM characterization (Figure 3). As can be seen from Figure 3, long ramified filaments are dominant, and quasi-spherical NPs are seldomly observed. The formation of two different morphologies confirmed both by TEM (Figure 2) and SEM studies (Figure 3) for the ablation using a Bessel beam could be tentatively explained using the inherent characteristics of a Bessel beam’s focusing geometry. During the focusing, the axicon provides a longer depth of focus with a sharp central intensity surrounded by concentric rings. The two distinct morphologies, spherical and filament-like, could be related to the different conditions created in these different zones. The SEM studies of the ablated target prove this statement since they reveal that melting occurred in the region of the outer rings as well, forming the elongated structures (Supporting Information File 1, Figure S4 and Figure S5). More detailed analysis of SEM images (Supporting Information File 1, Figure S4h,i) of the target exposed to the Bessel beam reveals the preferential formation of elongated, twisted, and branched structures, which might give rise to the nanofilaments observed in the colloid. Further confirmation of the participation of the outer rings in the processes of ablation and formation of elongated nanostructures is provided in Figure S5 (Supporting Information File 1). A clear ring-like pattern in the region of the outer rings is observed (Supporting Information File 1, Figure S5b), and the magnified image of the area exposed to irradiation from the outer rings (Supporting Information File 1, Figure S5c) shows individual twisted nanofilaments. This observation confirms the participation of the outer rings in nanofilament formation and proves melting of the target by the outer rings. In contrast, in the area exposed to the central lobe, the ablation features are similar to those of Gaussian beam ablation, shown for comparison in Supporting Information File 1, Figure S5e,f.

Furthermore, the simultaneous fabrication of NPs by the central lobe and a modification in the field provided by the outer rings of the Bessel beam should be considered when discussing the morphology peculiarities of the formed NPs. At later stages of the process, the outer rings might be also involved in nucleation and growth of nanoparticles by trapping the produced seeds and guiding their aggregation in distinct directions depending on the material and laser parameters. As shown previously [33], contrary to conventional optical traps using Gaussian beams, Bessel beam traps exhibit significant gradients only in the transverse directions, which leads to particle confinement in two dimensions with a scattering force in the propagation direction [34].

Formation of NPs in liquid media during laser ablation is known to be a complex phenomenon. A typical mechanism assumes that the ablation process starts with absorption of the laser pulse by a target during first tens of nanoseconds, followed by electron–phonon relaxations, plasma formation, shockwave propagation, and generation of CBs. The CBs expand for a few hundreds of microseconds and, after that, start oscillating and finally collapse. In case of ablation with Bessel or annular beams, the peculiarities of the energy distribution will influence plasma and CB shape, as well as parameters and dynamics of NP formation processes.

The structure of the Bessel beam implies that plasma formation would occur as a result of the central lobe interacting with the target, while the concentric rings, which have the laser energy distributed over a larger area may act as potential wells and can result in confinement of plasma and CB propagation, CB oscillations, and the reflection of shockwaves [29]. The confinement and reflection effects are known to result in a plasma compression and, consequently, plasma temperature and electron density increase [35–37]. A reflected shockwave also causes perturbations in the medium, thus, influencing the electron density distribution in the plasma. Furthermore, the confinement of the plasma plume slows down thermalization and cooling of the plasma, changing the conditions for the nucleation and growth of the nanoparticles [37]. The shockwave dynamics also have an impact on the CBs’ size, pressure, and oscillations which are the parameters influencing the morphology and structure of the forming NPs.

The characteristic structure of a Bessel beam also impacts the plasma shape and induces pressure and temperature gradients. This influence has been demonstrated in [26], where ultrafast laser ablation with radially polarized Bessel beams created a cylindrical plasma. The non-uniform energy distribution in the laser beam interacting with the target resulted in strong gradients of pressure and temperature in radial direction, which explained the formation of elongated pillar-shaped nanostructures on the target surface. For ablation in a liquid, the interaction of non-Gaussian beam field patterns with a target will further impact the ablation processes.

The dynamics and conditions in CBs are also affected by the laser beam shape and can be responsible for the observed nanofilaments. Although the CB dynamics in laser ablation with Bessel beams is yet to be studied, it can be assumed that a different pattern of the laser energy distribution on the target surface would vary the pressure at the laser–target interface [29], as well as influence CB evolution. These parameters strongly affect size and morphology of the forming NPs. In general, the dynamics of the CBs [38] is described by a modified Rayleigh–Plesset equation [39], which considers the pressure and size of CBs depending on parameters of both liquid (surface tension, density, viscosity, and temperature) and laser (pulse duration, fluence, and beam shape). A change of the incident beam pattern will therefore change the temperature and pressure inside the CBs and influence the bubble oscillations. We consider that the formation, evolution, and collapse of CBs are influenced by the Bessel beam field distribution and are different in comparison with the cases of annular and Gaussian focusing.

**Figure 4:**
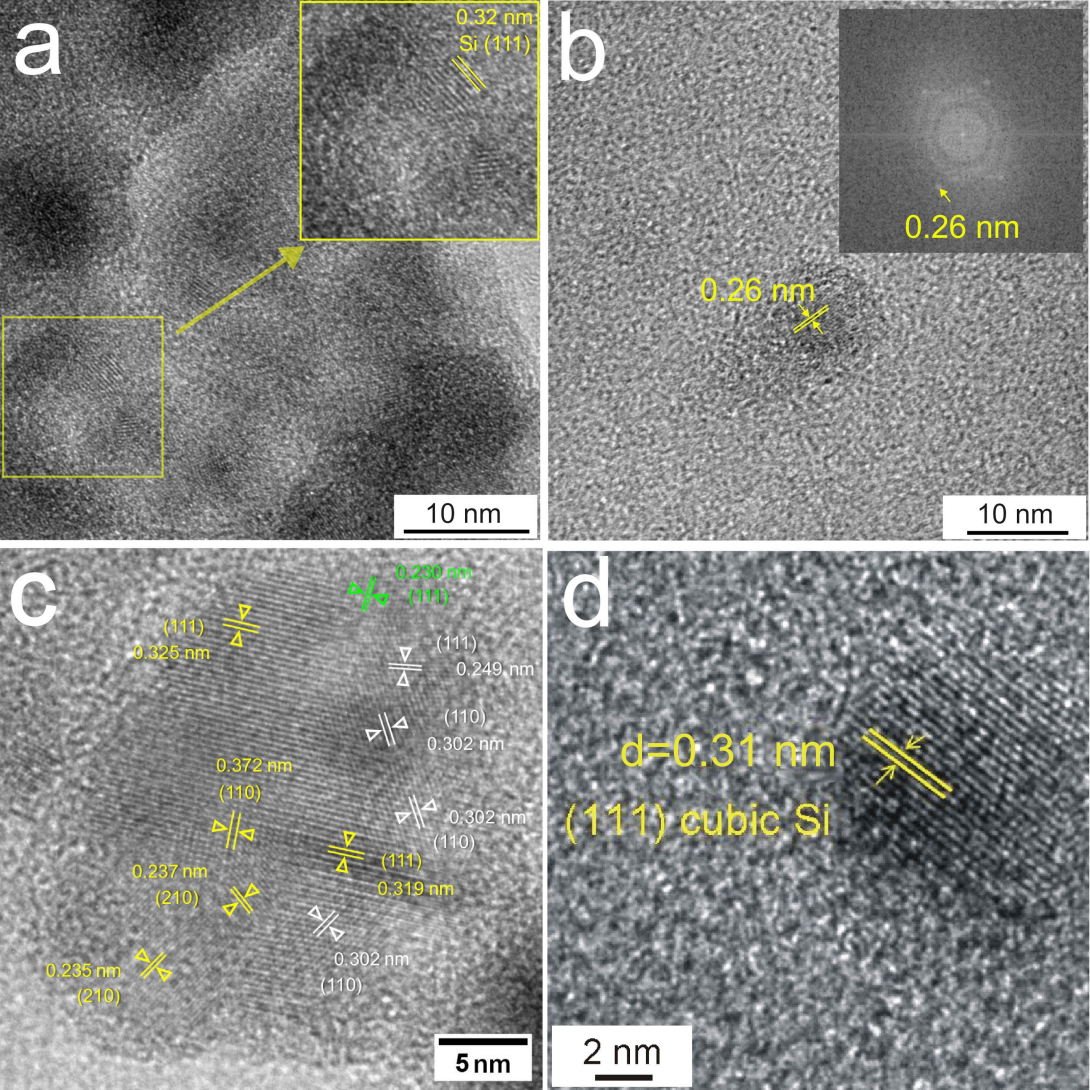
HRTEM images of the particles prepared by laser ablation of a Si wafer with (a, b) Bessel, (c) annular, and (d) Gaussian laser beams with interplanar spacings, which can be attributed to the planes of cubic Si, cubic SiC, and silicon oxide phases.

The inner structure of the prepared NPs was determined from HRTEM images. Figure 4a,b and Figure 4c show representative HRTEM images of NPs formed by laser ablation with Bessel and annular beams, respectively. The inner structure of the Si NPs prepared using a Gaussian profile has been studied in our previous work [7] and is shown in Figure 4d. The results showed that cubic Si is the dominant phase if the ablation is performed in ethanol under these conditions. It should be noted that particles formed by laser ablation with annular beams (Figure 4c) are also crystalline and have a diamond-like cubic crystal structure, where the interplanar spacings correspond to the reflections from (111) planes of cubic Si (space group *Fd−3m*). Some particles shown in Figure 4c have measured interplanar distances (0.25 nm) in agreement with the reflections from the (111) planes of cubic 3C-SiC [40]. The formation of silicon carbide during laser ablation of silicon in ethanol has been observed in our previous work [10] and can be explained by chemical reactions of the ejected silicon species with products of ethanol decomposition or ethanol molecules. In [10], this mechanism has been supported by the results of optical emission spectroscopy of the generated plasma, which showed the presence of silicon species, including atomic (390.55 nm) and ionic (385.61, 413.10, and 505.61 nm) lines, as well as emission of the ethanol decomposition products (C_2_ Swan band at 516.52 nm) with significantly lower contribution than that from Si NPs [10].

Some admixture of a hexagonal silicon dioxide phase was also observed, but mainly at the interface of particle agglomerates. However, as it could be concluded from the structural analysis of the particles formed during laser ablation with the Bessel beam, only some of the small nearly spherical NPs are crystalline and mainly composed of cubic silicon or silicon carbide phases. Additional interplanar distances in the HRTEM images could be indexed to either the (210) planes of Si or the (110) planes of hexagonal SiC. The observations support the assumption that cubic crystal structures are more probable and stable than the hexagonal ones. More details of the HRTEM analysis can be found in the Supporting Information File 1, Figure S1, and Tables S1 and S2.

### Optical characterization

**Figure 5:**
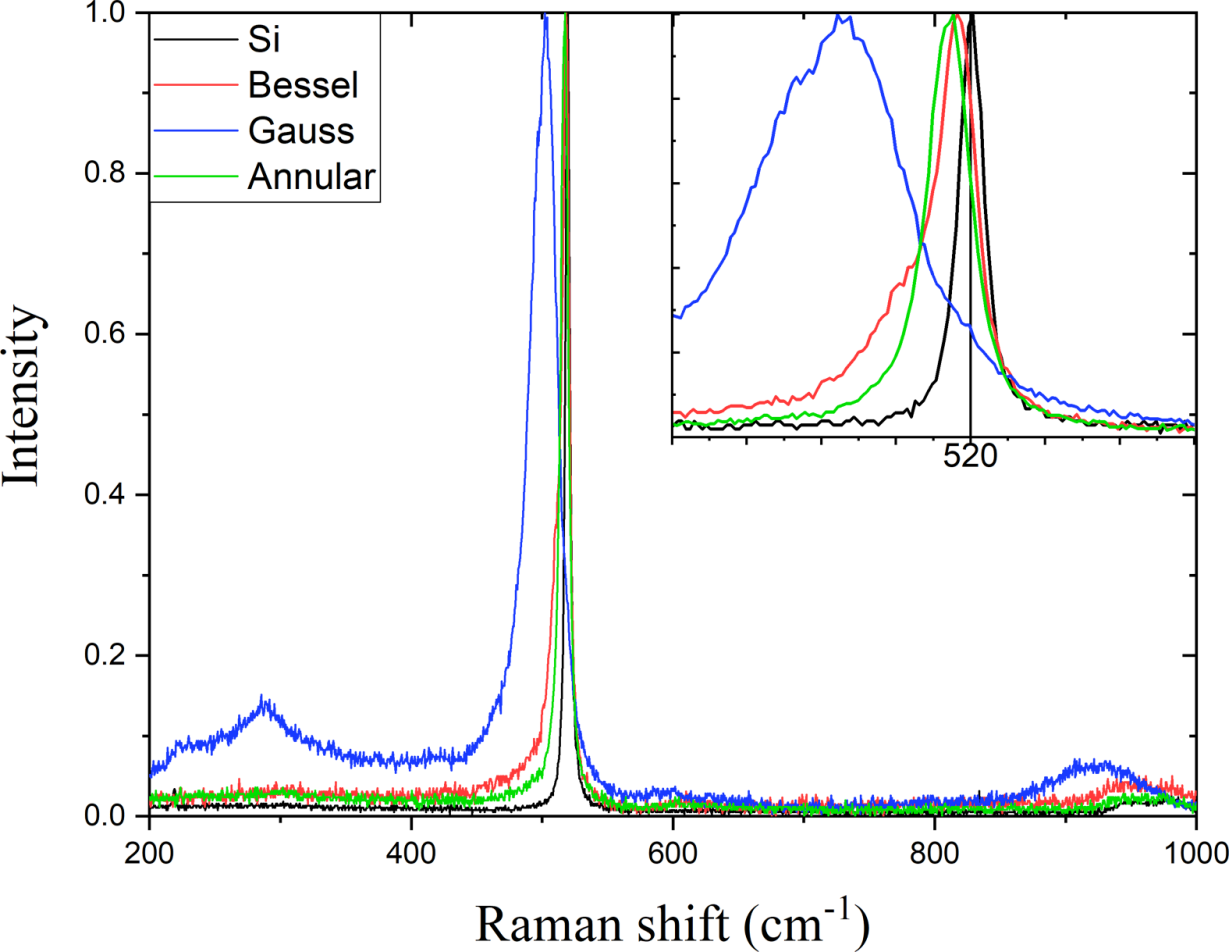
Raman spectra of Si samples obtained using laser beams with Gaussian, Bessel, and annular profiles.

The optical properties of Si NPs dispersed in ethanol were investigated using Raman and UV–vis spectroscopy. Initially, Si NPs obtained using Bessel, annular, and Gaussian laser beams were characterized by Raman spectroscopy, and their Raman spectra (vibrational modes) were compared with the vibrational modes of the unablated bulk Si target. Figure 5 presents the obtained Raman spectra. In general, when bulk crystalline Si is transformed into Si NPs, an amorphous phase in Si nanocomposites can be formed [41]. Usually, amorphous Si Raman modes are shifted to lower wavenumbers compared to crystalline Si. For example, according to [12], Si NPs exhibit both crystalline and amorphous Raman peaks at 517 and 477 cm^−1^, respectively.

All major peaks in the Raman spectrum are attributable to the spectrum of silicon. The peaks are located in the ranges of 502–518, 284–285, and 925–950 cm^−1^, which correspond, respectively, to the TO, 2TA, and 2TO phonon modes of silicon structures [10]. The positions of the peaks are, however, different from those in the bulk silicon spectrum. The major peaks are downshifted for all experimental conditions used, caused by size effects, lowering of crystallinity of the silicon samples, or amorphization.

An effect of laser beam shape has been also observed in the Raman spectra analysis. Though Si NPs generated from the crystalline Si target using laser beams of different shapes exhibit all above Raman modes, the positions and intensities of these Raman modes are slightly different. This reflects a difference in dimensions, number density, crystallinity, and probably morphology of Si NPs formed by using laser beams with different profiles. The corresponding asymmetric broadening of the Raman peaks toward lower wavenumbers indicates a possible contribution from the amorphous phase, with the intensity variation among them representing variations in the ratio of amorphous and crystalline phases. For example, the 2TA and 2TO phonon modes of Si NPs obtained by Gaussian laser beam ablation are higher in intensity in comparison with those of the samples produced using Bessel and annular profiles. It should be noted that the fractions of amorphous and crystalline phases can be determined by deconvolution of the Raman peaks profiles to estimate the crystallinity of Si NPs (Supporting Information File 1, Figure S6).

**Figure 6:**
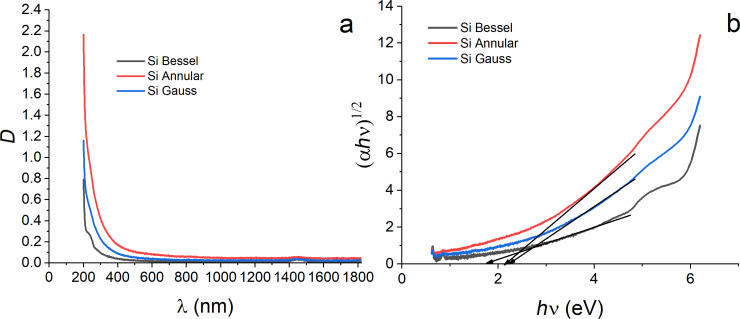
Optical properties of the Si NPs prepared by laser ablation of a Si target in ethanol using Bessel, annular, and Gaussian laser beams. (a) UV–vis absorption spectra and (b) Tauc plots.

The colloids synthesized using different laser beams also showed different optical properties, which were characterized using UV–vis absorption and PL spectroscopies. The absorption spectra of the Si colloids prepared using Gauss, Bessel, and annular profiles are shown in Figure 6. In all the spectra, absorption in the visible and near-infrared regions has been detected. However, as can be concluded from Figure 6a, the absorption cutoff wavelength differs for each studied sample, indicating different optical and electronic properties of the formed nanostructures. The variation of the optical properties can be caused by structural differences of the NPs, which is in agreement with the structural characterization discussed above.

More details regarding the differences in electronic and optical properties of the formed nanostructures can be obtained from the analysis of Tauc plots. A Tauc plot, that is the dependence of (α*h*ν)^1/^*^n^* on *h*ν, provides the information about the bandgap energies of the prepared Si NPs. Here, α, *h*, and ν are absorption coefficient, Planck constant, and frequency, respectively; *n* depends on the type of bandgap and can be 2 for indirect or 1/2 for direct bandgaps, respectively (Figure 6b). For silicon, indirect bandgap characteristics are typical; therefore, the value of *n* was chosen as 2. Note that silicon is known to have different crystal structures, whose optical properties may vary. Furthermore, size effects are also influencing the optical properties of the resulting NPs [42]. As shown in [42], the bandgap of nanocrystals increases with decreasing size due to quantum confinement. Accordingly, the estimated bandgaps for all the prepared colloids was found to be in the range of 1.8–2.2 eV, which is larger than the bandgap of bulk Si (1.12 eV) and can be attributed to the NPs size effects (Figure 6b). The Si NPs formed by the Bessel laser beam show the lowest bandgap (1.8 eV), whereas those obtained with the Gaussian and annular beams exhibited higher bandgap values (2.1 and 2.2 eV, respectively).

**Figure 7:**
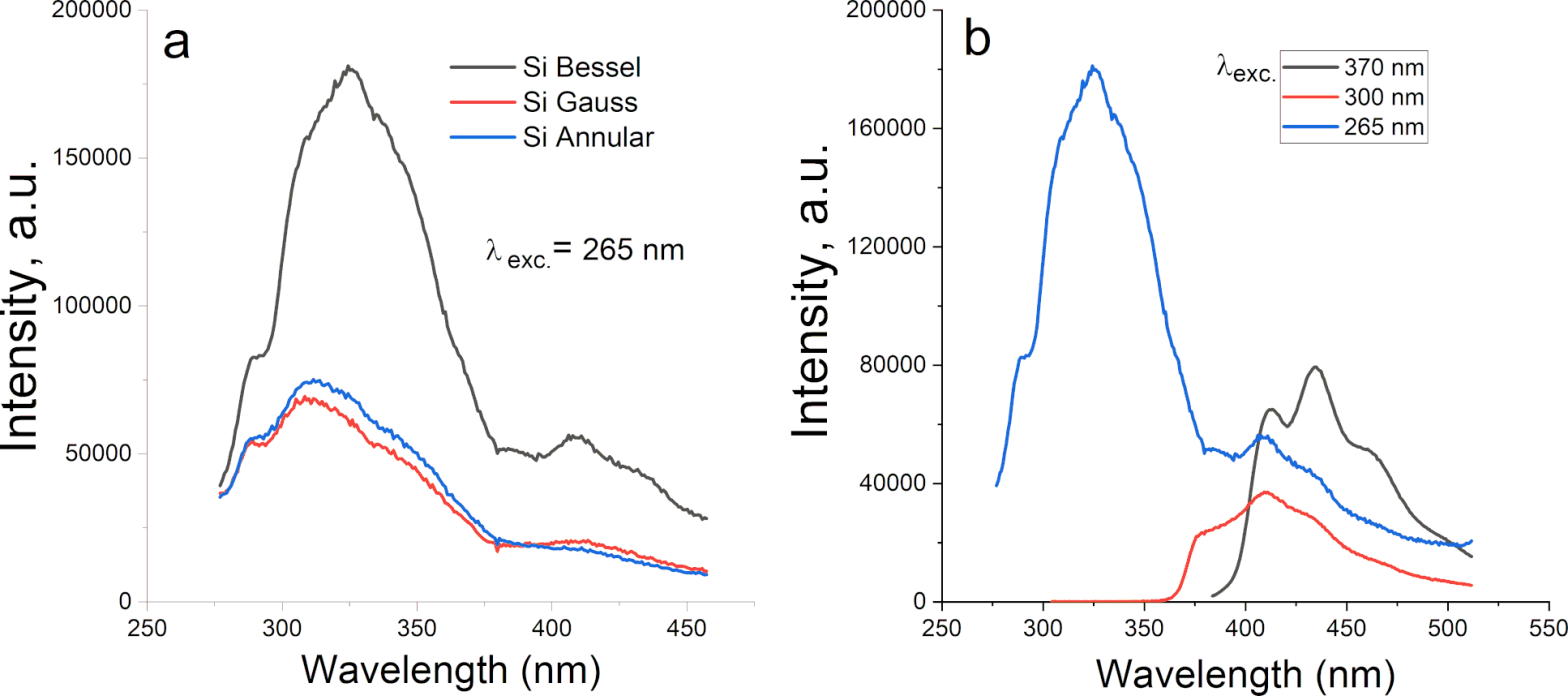
Photoluminescence spectra of (a) Si NPs prepared by Bessel, Gaussian, and annular laser beam ablation under excitation at 265 nm and of (b) Si NPs prepared by Bessel laser beam ablation under excitation at 265, 300, and 370 nm.

The effects of laser beam shape on the luminescence properties of Si NPs were studied via PL spectroscopy, with excitation at 265, 300, and 370 nm. The corresponding spectra are summarized in Figure 7. All samples demonstrated emission in the range of 300–500 nm. However, the characteristics of these emission bands deviate slightly for each sample. The emission in the UV range can be caused by the recombination of free excitons in a quantum confined system [43–45], while the blue and green emissions can be attributed to defect states or surface traps generated during the laser ablation process. For example, the observed photoluminescence can originate from the radiative recombination of photogenerated holes with electrons from the oxide-related defects on the surface of silicon nanoparticles [44,46]. The characteristic PL intensities are different for Si NPs prepared by laser ablation with different beam profiles, which can indicate variations among the generated defects during the formation of Si NPs.

The presence of defects in the Si structure confirmed by Raman and PL studies is a prerequisite for enhanced performance of the developed nanostructures in electrochemical processes occurring in supercapacitors or batteries [14]. The intrinsic defects act as intercalation sites during charge and discharge, facilitating the charge transfer processes [47–49]. As a result, parameters such as specific capacitance and cycling stability are enhanced [13].

In our case, high values of specific capacitance can be expected because of the developed surface of the silicon NPs prepared by laser ablation with the Bessel beam profile. Another application of the nanofilaments prepared using Bessel beams can be the electrode material of Li-ion batteries. Experiments on the preliminary evaluation of the electrochemical properties of the prepared nanomaterials for their potential application as a supercapacitor electrode material are in progress. In these experiments, the fabricated Si NPs are deposited onto a carbon cloth and used as working electrodes in the three-electrode scheme. As shown in [50], the application of carbon cloth as a support material in supercapacitors demonstrated a number of benefits from the mechanical and chemical stability of the carbon cloth material, which, together with its low cost and large surface area, make it especially promising for the flexible devices. The results of these experiments are the subject of upcoming works.

## Conclusion

In summary, laser beam shaping can be considered as an important parameter in the optimization of Si NPs synthesis in terms of morphology control of the produced NPs and as a viable approach to expand laser ablation capabilities in the field of material processing.

Nanocrystalline particles of silicon were synthesized by pulsed laser ablation in ethanol, using three different laser beam profiles. A Bessel beam was produced using an axicon, an annular profile was formed using a combination of the axicon and a converging lens, and a Gaussian profile was generated using solely the converging lens. A correlation of NP properties with the laser beam profiles was found. The beam profile was shown to be a crucial factor significantly influencing the morphology of the nanostructures produced. The conditions generated during laser ablation using a beam with Bessel profile favored the production of nanostructures having elongated filament-like morphology. The analysis of the Raman and PL spectra of the synthesized NPs allowed for conclusions about the defects formed during synthesis. PL emission was observed in the range of 300–500 nm, which, for Si nanostructures, can be explained by recombination of photogenerated carriers and the presence of defects in the structure. The synthesized colloidal Si NPs can be collected directly in liquid onto substrates for further applications, such as electrode materials in supercapacitors and batteries.

## Supporting Information

File 1 Additional experimental results and analysis of the produced Si NPs.

## Data Availability

Data generated and analyzed during this study is available from the corresponding author upon reasonable request.
