# Supplementary material for: Influence of laser beam profile on morphology and optical properties of silicon nanoparticles formed by laser ablation in liquid
Source: Beilstein J Nanotechnol. 2025 Sep 4;16:1533–44. doi: 10.3762/bjnano.16.108 (PMC12415900; doi:10.3762/bjnano.16.108)
Supplement: File 1 — Additional experimental results and analysis of the produced Si NPs. [file Beilstein_J_Nanotechnol-16-1533-s001.pdf]

BEILSTEIN JOURNAL OF NANOTECHNOLOGY

Supporting Information

for

Influence of laser beam profile on morphology and optical properties of silicon nanoparticles formed by laser ablation in liquid

Natalie Tarasenka, Vladislav Kornev, Alena Nevar and Nikolai Tarasenko

Beilstein J. Nanotechnol. **2025,** *16,* 1533–1544. doi:10.3762/bjnano.16.108

Additional experimental results and analysis of the produced Si NPs

License and Terms: This is a supporting information file under the terms of the Creative Commons Attribution License (https://creativecommons.org/licenses/by/4.0). Please note that the reuse, redistribution and reproduction in particular requires that the author(s) and source are credited and that individual graphics may be subject to special legal provisions.
The license is subject to the *Beilstein Journal of Nanotechnology* terms and conditions: (https://www.beilstein-journals.org/bjnano/terms)

Transmission electron microscopy

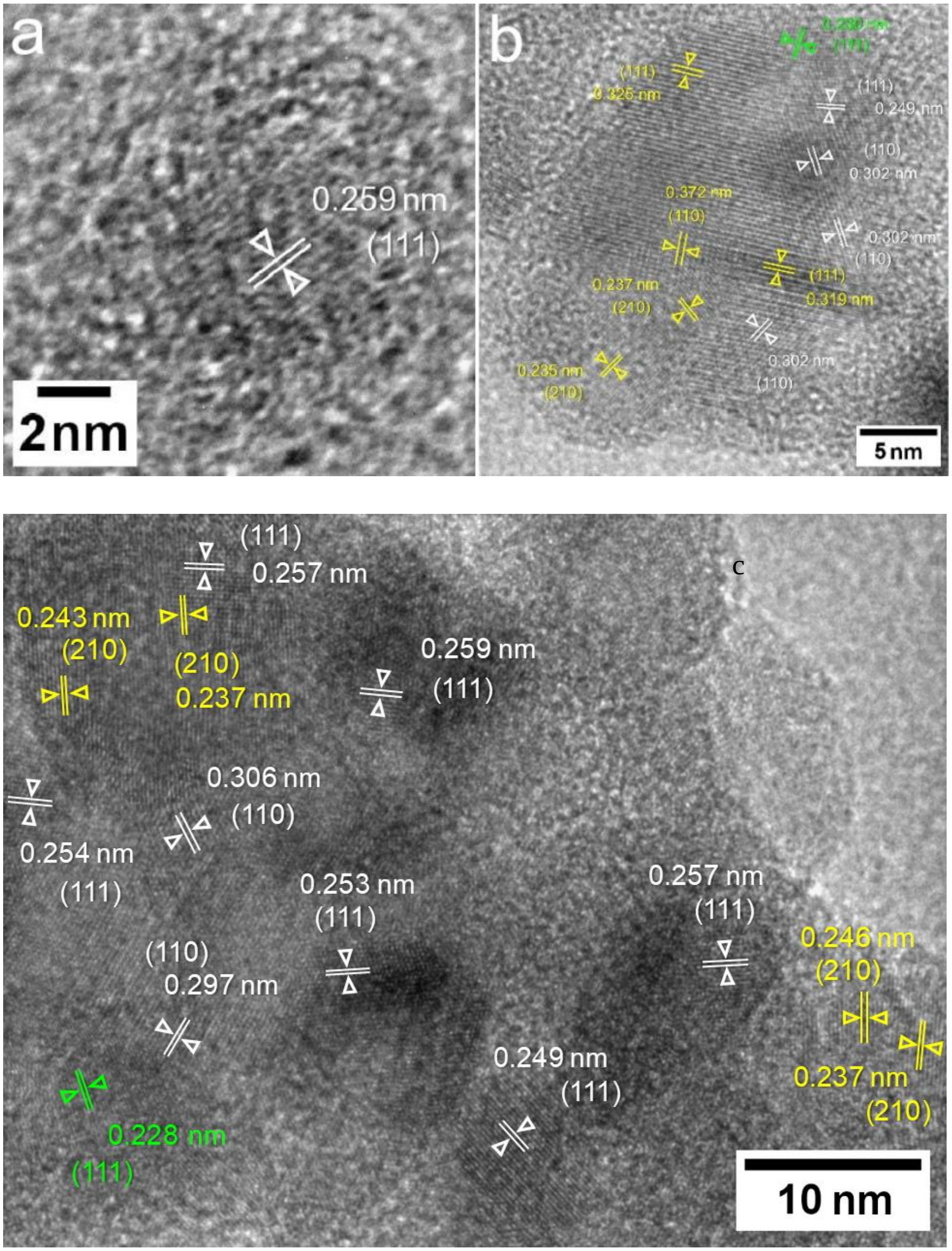

Figure S1: HRTEM images of separate particles prepared by laser ablation of Si wafer with the Bessel (a) and annular (b,c) laser beams indicating the interplanar spacings**,** which can be attributed to the planes of cubic Si, cubic SiC**,** and silicon oxide phases.

Table S1: Determination of the interplanar spacings.

Color highlighting	Material	Crystal structure and space group	$d_{measured}$, nm	d_{calc}, nm	Miller indices (hkl)	Δ*d*, nm
yellow	Si	cubic *Fd*−3*m*	0.237	0.243	(210)	±0.006
			0.246	0.243	(210)	±0.003
			0.243	0.243	(210)	±0.000
			0.235	0.243	(210)	±0.008
			0.319	0.314	(111)	±0.005
			0.325	0.314	(111)	±0.011
			0.372	0.385	(110)	±0.013
green	SiO_2	hexagonal $P3_221$	0.228	0.224	(111)	±0.004
			0.230	0.224	(111)	±0.006
white	SiC	cubic *F*−43*m*	0.297	0.307	(110)	±0.010
			0.302	0.307	(110)	±0.005
			0.249	0.251	(111)	±0.002
			0.253	0.251	(111)	±0.002
			0.254	0.251	(111)	±0.003
			0.306	0.307	(110)	±0.001
			0.257	0.251	(111)	±0.006
			0.259	0.251	(111)	±0.008

Table S2: Phase composition of the NPs prepared using different beam profiles.

Phase composition, Miller indices (hkl)	Beam profile	
	Bessel	annular
Si	(111)	(111), (110), (210)
SiC	(111)	(111), (110)
SiO_2	—	(111)

Size distributions of the NPs

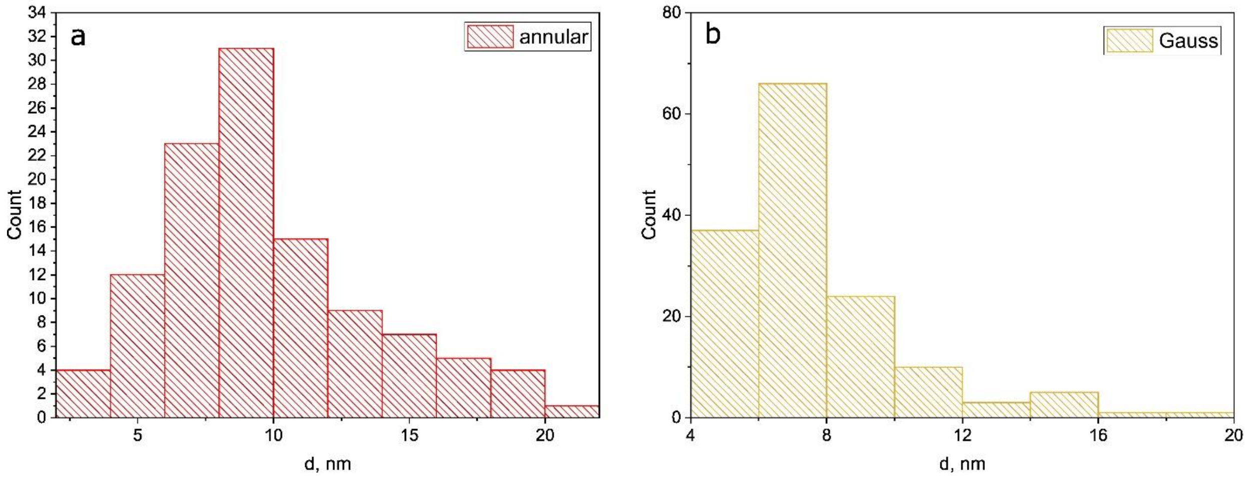

Figure S2: Size distribution of the NPs prepared by laser ablation of Si target in ethanol using (a) annular and (b) Gaussian beam profiles.

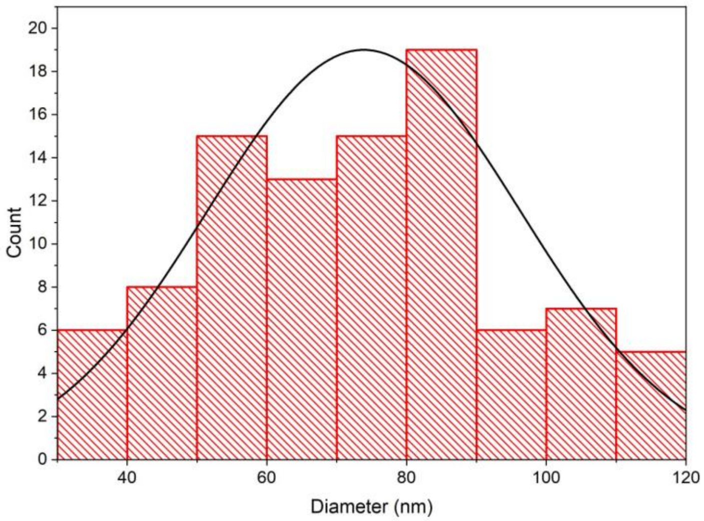

Figure S3: Histogram of diameter distribution of nanofilaments prepared by laser ablation of a Si target in ethanol using a Bessel beam profile. The size distribution was determined with the aid of ImageJ software. The distribution was nearly normal (Gaussian) type.

Scanning electron microscopy of the ablated Si target

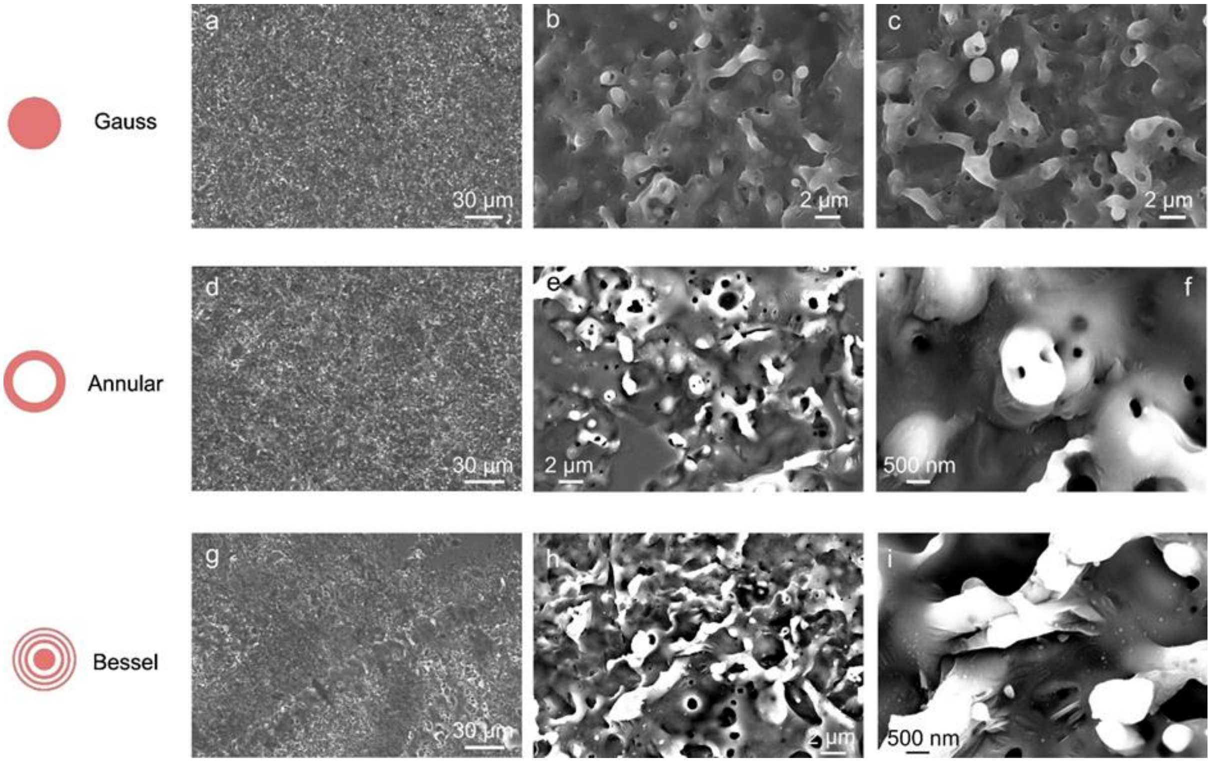

Figure S4: SEM characterization of Si targets after laser ablation using (a–c) Gauss, (d–f) annular, and (g–i) Bessel beam profiles. The SEM images are presented with different magnifications.

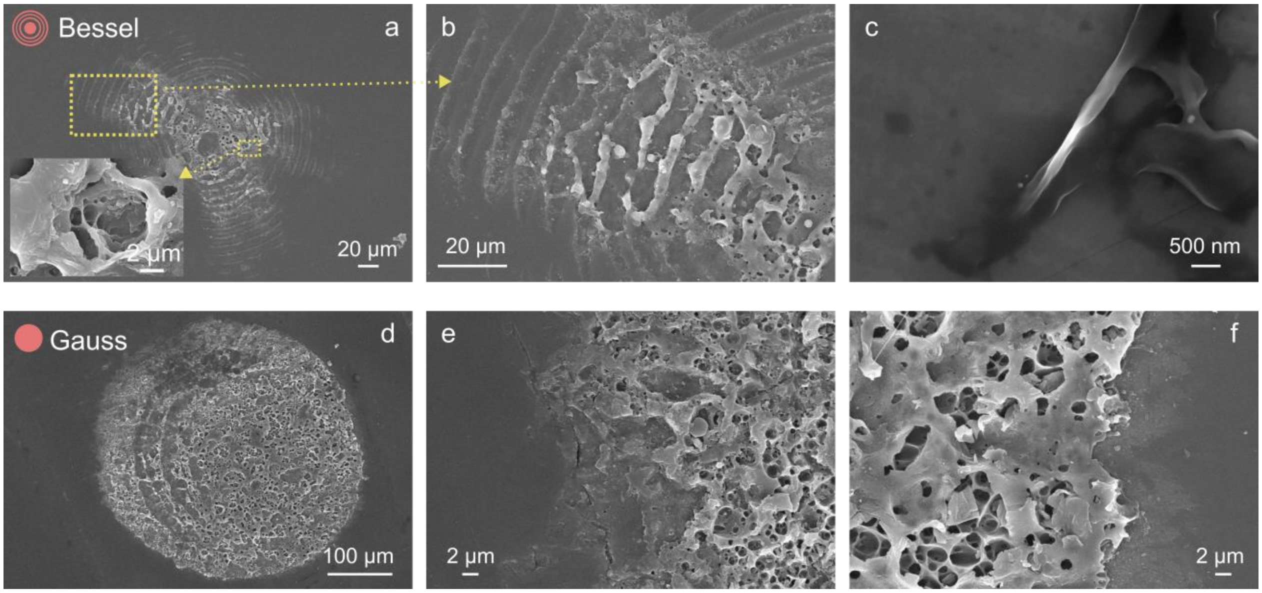

Figure S5: SEM images of Si targets exposed to a single laser pulse of (a–c) Bessel and (d–f) Gaussian beams. Panel c shows individual twisted filaments forming on the surface in the area of outer-ring irradiation.

Raman spectra of Si NPs

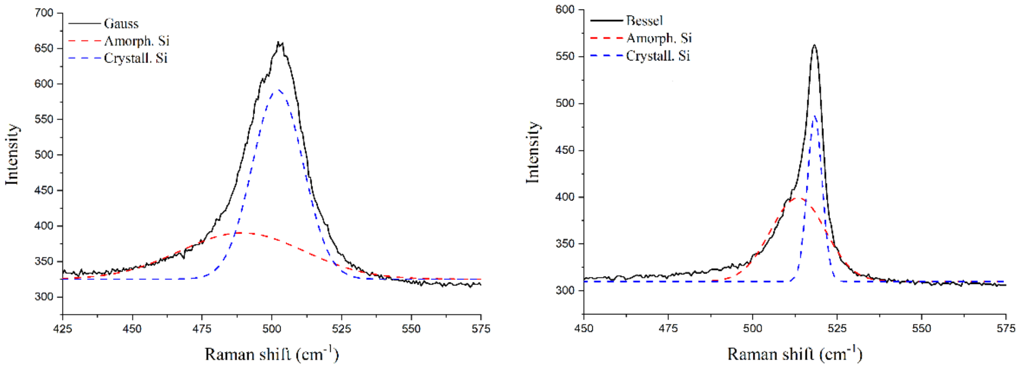

Figure S6: Raman spectra deconvolution into Gaussian (crystalline, blue line) and asymmetric (amorphous, red line) components for Si NPs prepared by laser ablation of Si in ethanol using Gauss and Bessel laser beams.

Deconvolution of the Raman spectra in Figure S6 in the range from 400 to 540 cm^{-1} into Gaussian and asymmetric components based on the method described in [1] enables the estimation of the partial volumes of crystalline and amorphous phases in the prepared Si samples. In all cases, the best fit results were obtained using two components. The relative content of the crystalline phases can be estimated as 0.77 and 0.67 for samples prepared with Bessel and Gauss laser beams, respectively.

References

1. Neimash, V.; Poroshin, V.; Shepeliavyi, P.; Yukhymchuk, V.; Melnyk, V.; Kuzmich, A.; Makara, V.; Goushcha, A. O. *J. Appl. Phys.* **2013,** *114*, 213104. doi:10.1063/1.4837661
